# Uncovering ferroelectric polarization in tetragonal (Bi_1/2_K_1/2_)TiO_3_–(Bi_1/2_Na_1/2_)TiO_3_ single crystals

**DOI:** 10.1038/s41598-019-55576-y

**Published:** 2019-12-17

**Authors:** Yuuki Kitanaka, Yuji Noguchi, Masaru Miyayama

**Affiliations:** Department of Applied Chemistry, School of Engineering, The University of Tokyo, 7-3-1 Hongo, Bunkyo, Tokyo 113-8656 Japan

**Keywords:** Ferroelectrics and multiferroics, Inorganic chemistry

## Abstract

We report the robust ferroelectric properties of (1 − *x*)(Bi_1/2_Na_1/2_)TiO_3_–*x*(Bi_1/2_K_1/2_)TiO_3_ (*x* = 33%) single crystals grown by a top-seeded solution growth process under a high oxygen-pressure (0.9 MPa) atmosphere. The sample exhibit a large remanent polarization of 48 μC/cm^2^ and a sizeable piezoelectric strain constant of 460 pm/V. Neutron powder diffraction structural analysis combined with first-principles calculations reveals that the large ferroelectric polarization comparable to PbTiO_3_ stems from the hybridization between Bi-6*p* and O-2*p* orbitals at a moderately negative chemical pressure.

## Introduction

A versatile structural framework of ABX_3_ perovskites delivers a wide variety of electronic properties^1,2^ such as superconductivity^3–5^, dielectric permitivity^6–9^, ionic conductivity^10–13^ and magneto-electric effects^14,15^. These functionalities arise from electron correlations and/or electron-lattice coupling, which can be designed by a diverse combination of the constituent elements^2,16,17^. For the A-site atoms, Bi is of considerable importance from fundamental and practical points of view, thanks to the strong interactions between Bi-6*s*(*p*) and X-*p* orbitals. For photovoltaic devices, Bi-based halides show a high efficiency derived from antibonding Bi-6s states near the valence band maximum^18–20^. Bismuth ferrite BiFeO_3_ is a rare multiferroic material at room temperature exhibiting a coupling between ferroelectric and antiferromagnetic orders, where the covalency arising from the Bi-6*p* and O-2*p* hybridization gives rise to a robust ferroelectric polarization^21–25^.

A combination of Bi with alkali metals on the A-site enables to accommodate higher-valent cations such as Ti^4+^ on the B site. Bismuth potassium titanate (Bi_1/2_K_1/2_)TiO_3_ [BKT] has ferroelectricity in a tetragonal structure (space group *P*4*mm*) at room temperature, which has attracted much attention as a lead-free piezoelectric material^26^. The solid solution with rhombohedral (Bi_1/2_Na_1/2_)TiO_3_ [BNT; space group *R*3*c*]^26,27^, i.e., the BKT–BNT system, displays a morphotropic phase boundary (MPB), where the ferroelectric structure changes dramatically and the piezoelectric activity is maximal^28–33^. There have been intense efforts on BKT-based ceramics to replace lead zirconate titanate (PZT) currently used for various applications^34,35^.

By contrast, few studies on BKT-based single crystals have been performed to date because of the difficulty in growing high-quality samples. At a temperature above 1300 K, BKT undergoes a thermal decomposition^36^, which makes the crystal growth difficult; to the best of our knowledge, the preparation of BKT crystals has never been achieved. Although some studies have reported the crystal growth of BKT-based solid solutions^37–39^, these crystals suffer from the problems arising from point defects. Owing to a high vapor pressure, Bi is apt to evaporate from the lattice leaving a vacancy of Bi (*V*_Bi_”’), which is accompanied by the formation of oxygen vacancy (*V*_O_^••^)^40^. In addition, an oxidation treatment is required for as-grown samples but increases leakage currents to some extent because of p-type conduction, which prevents us from applying an electric field (*E*)^41,42^. Moreover, *V*_O_^••^ tends to accumulate at ferroelastic domain walls, which are strongly pinned and eventually clamped even under high fields^43,44^. For revealing the ferroelectric nature, it is desirable to develop a high-quality single crystal with a low concentration of *V*_Bi_”’ where external fields can switch spontaneous polarization (*P*_s_).

In this study, we report a growth of high-quality BKT-based single crystals exhibiting a complete switching of *P*_s_, employing the top-seeded solution growth (TSSG) method under high-oxygen-pressure (high-*P*o_2_) atmosphere^45,46^. We chose a BKT-rich tetragonal phase in the BKT–BNT system. Our process enables us to obtain relatively large and high-performance single crystals with a large *P*_s_ of 48 *μ*C/cm^2^ and a high piezoelectric strain constant (*d*_33_) of 460 pm/V. Structural analysis combined with first-principles calculations reveals that the robust ferroelectric polarization and resultant high *d*_33_ stem from the orbital interaction between Bi-6*p* and O-2*p* at a moderate chemical pressure.

## Results

**Figure 1:**
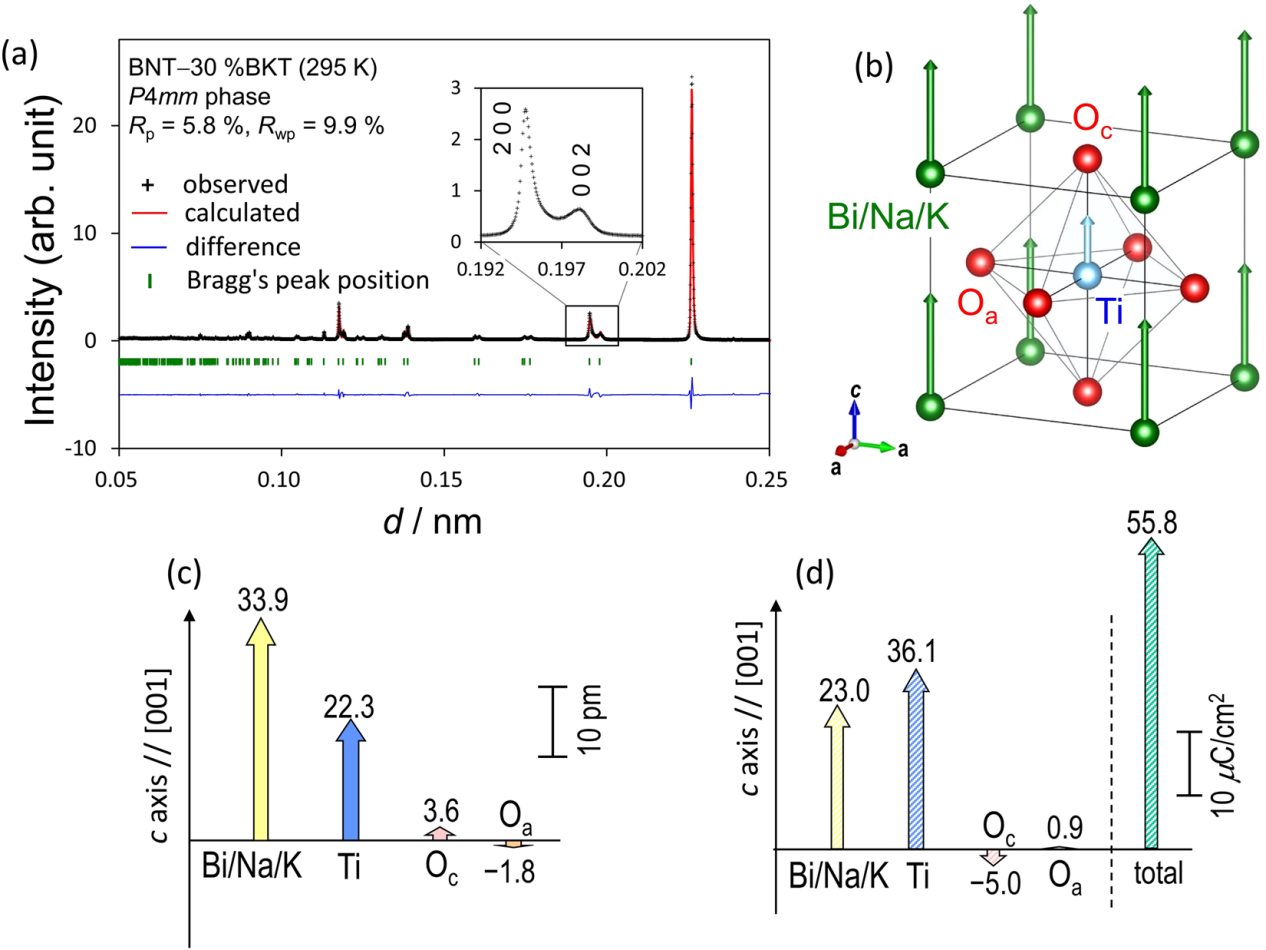
Refined crystal structure of tetragonal BNT-BKT. (**a**) Fitting results of the Rietveld refinement analysis of the NPD data for BNT–BKT (*x* = 30%). (**b**) Refined crystal structure for the tetragonal *P*4mm phase of BNT–BKT (*x* = 30%). (**c**) Polar displacements and (**d**) dipole moments of the constituent atoms along the *c* axis of the refined tetragonal *P*4mm phase of BNT-BKT (*x* = 30%).

Neutron diffraction data were collected^47^ for (1 − *x*)BNT–*x*BKT powders (*x* = 30–35%) prepared by solid-state reaction, and the powders were found to have a tetragonal *P*4*mm* structure. Figure 1(a) shows the fitting result of the Rietveld analysis for *x* = 30% measured at 295 K along with the calculated profile, the difference in intensity, and the peak positions. Our analysis leads to a profile reliability (*R*_p_) factor of 5.8% and a weighted *R*_p_ (*R*_wp_) factor of 9.9%. All reflections are indexed by a perovskite unit cell in tetragonal *P*4*mm* symmetry indicating a clear splitting of, e.g., 200 and 002 reflections, which is due to a tetragonal strain (*c*/*a*) of 1.016. The refined crystal structure with the atomic displacements is illustrated in Fig. 1(b) and the detailed structural parameters are summarized in Supplementary Information I.

Figure 1(c) shows the displacements of the constituent atoms along the *c* (polar [001]) axis. The off-center displacements are estimated from the hypothetic paraelectric structure, whose origin is set to the center of mass of the oxygen octahedron. The displacement of the A-site atoms (Bi_0.50_Na_0.35_K_0.15_) is as large as 0.034 nm, which is about twice that of the B-site atom (Ti). Figure 1(d) depicts the electric dipole moments of the constituent atoms estimated from the off-center displacements [Fig. 1(c)] and the averaged effective charges obtained by the DFT calculations. The cooperative displacements of the A- and B-site atoms lead to the parallel dipole moments of *p*_A_ = 23.0 μC/cm^2^ for the A-site atoms and *p*_B_ = 36.1 μC/cm^2^ for the B-site one. This dipole configuration provides a large *P*_s_ of 55.8 μC/cm^2^.

**Figure 2:**
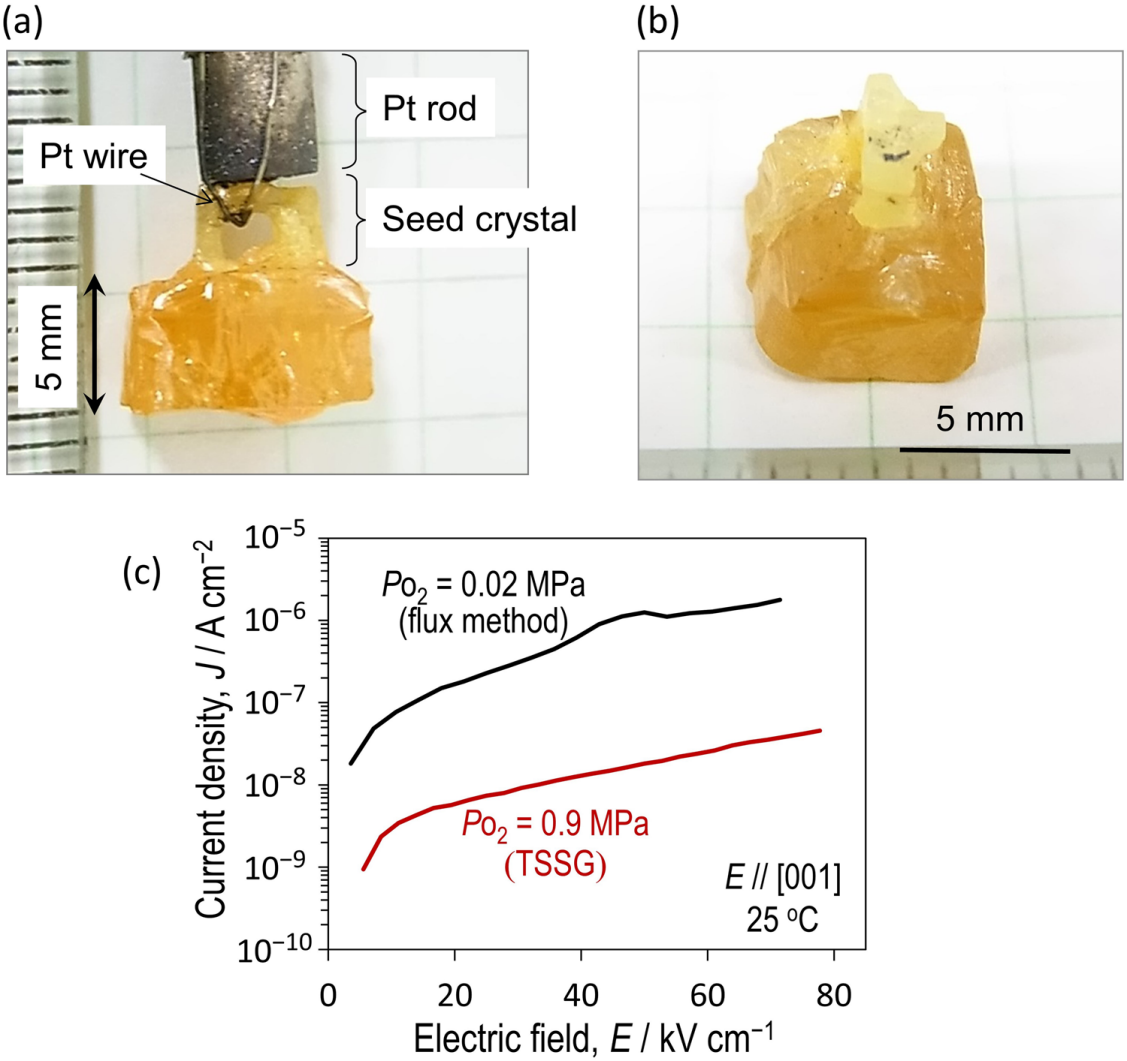
Grown crystal exhibiting a low leakage current. (**a**) Side and (**b**) perspective view of an as-grown single crystal of BNT-BKT (*x* = 33%) grown by the TSSG method at a *P*_O2_ of 0.9 MPa. (**c**) Leakage current densities along the [001] direction of BNT-BKT (*x* = 33%) single crystals grown at a *P*_O2_ of 0.02 and 0.9 MPa.

Our crystal-growth process provides a high-quality single crystal of *x* = 33% [Fig. 2(a,b)]. The crystal is transparent in yellow color with dimensions of 6 × 6 × 5 mm^3^. X-ray diffraction analysis shows that the crystallographic orientation coincides with that of the seed crystal, indicating an epitaxial growth from the seed. Figure 2(c) shows the leakage current density (*J*) at 298 K as a function of electric field (*E*) applied along [001], where the data of the crystal grown in the air (*P*o_2_ = 0.02 MPa) is also shown. The crystal grown at *P*o = 0.9 MPa exhibits a lower *J* by 1–2 orders of magnitude than that at *P*o_2_ = 0.02 MPa.

**Figure 3:**
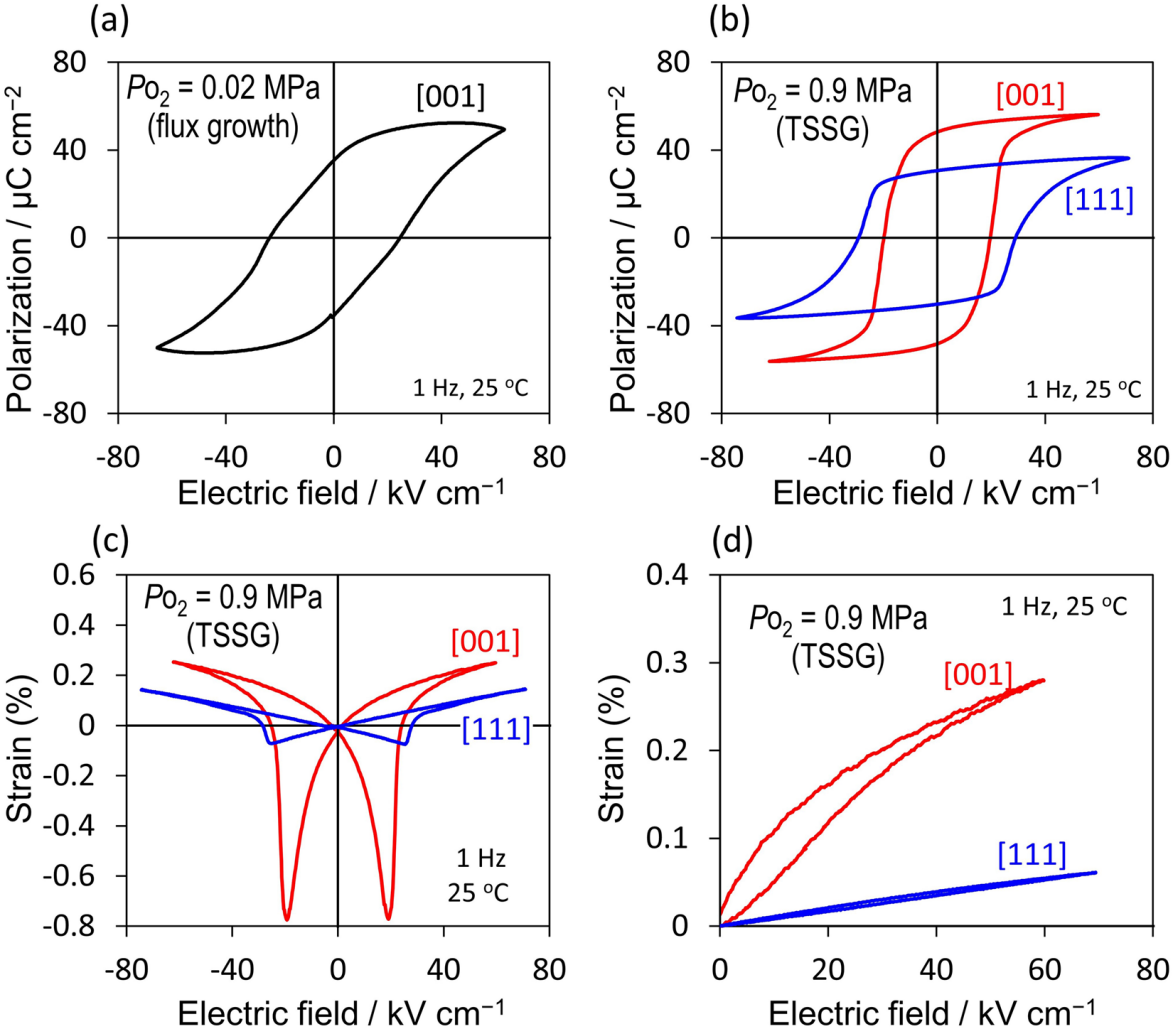
Polarization and strain properties. (**a**,**b**) Polarization properties of BNT-BKT (*x* = 33%) single crystals grown at (**a**) *P*_O2_ = 0.02 MPa and (**b**) *P*_O2_ = 0.9 MPa. The index beside each curve indicates the crystallographic direction of the polarization measurements. (**c**,**d**) Strain properties of BNT-BKT (*x* = 33%) single crystals grown at *P*_O2_ = 0.9 MPa under (**c**) bipolar and (**d**) unipolar electric fields along the [001] and [111] directions.

Figure 3 shows the polarization (*P*) and strain (*S*) properties at 298 K. The crystal (*P*o_2_ = 0.02 MPa) [Fig. 3(a)] exhibits a remanent polarization (*P*_r_) of 32 μC/cm^2^ and a coercive field (*E*_c_) of 23 kV/cm; because this crystal displays a high *J* of the order of ~10^−6^ A/cm^2^ at high fields, the leakage currents are comparable to the polarization-switching currents in the *P-E* measurements, leading to a blunted response. By contrast, the crystal (*P*o_2_ = 0.9 MPa) [Fig. 3(b)] features a well-saturated loop with a large *P*_r_ of 48 μC/cm^2^ and a low *E*_c_ of 18 kV/cm. The blue line in Fig. 3(b) indicates the *P*-*E* loop along [111] with a *P*_r_ of 30 μC/cm^2^. Provided that the application of *E* along [111] can lead to a complete switching of *P*_s_ (//[001]), the measurement along [111] yields *P*_s_/√3 as a *P*_r_ (32.2 μC/cm^2^), which is close to the observed one (30 μC/cm^2^). Our measurements along [111] and [001] lead to a *P*_s_ of 50–52 μC/cm^2^, which is in good agreement with that (55.8 μC/cm^2^) determined by the NPD analysis with the DFT calculations.

Figure 3(c) shows the *S*-*E* curves along [001] and [111]. The crystal (*P*o_2_ = 0.9 MPa) displays a clear butterfly loop in the bipolar measurement; a strain reaches up to ~0.3% along [001], which is larger than that along [111]. A piezoelectric strain constant (d^*^) estimated from the slope of the unipolar curve at *E* < 5 kV/cm is as high as 460 pm/V along [001], which is comparable to that^34^ for commercial PZT ceramics. The bipolar curve along [001] exhibits a large negative *S* of ~−0.8% at *E* = *E*_c_ (20 kV/cm), whereas that along [111] has a negative *S* that can be extrapolated from the slope. These results enable us to understand the mechanism of the polarization-switching dynamics under *E*. If the *P*_s_ vector is reversed by the 180° switching and a 90° domain structure does not participate in the process, the negative *S* is attributed solely to the converse piezoelectric effect. In this case, the slope of *S* with decreasing *E* remains constant until the 180°*-P*_s_ switching starts, and the negative *S* can be expected from d^*^. Provided that the polarization reversal proceeds through the 90°*-P*_s_ switching, i.e., the successive rotation of *P*_s_ by 90° mediated in a 90° domain state, this process is accompanied by a change in crystallographic configuration from *E* // **c** to *E* // **a** in each domain. Assuming that the state at *E* = *E*_c_ has a 90° domain structure composed of the domains in the *E* // **c** and *E* // **a** configurations, we estimate an averaged *S* of –(*c* − *a*)/2*a* to be −0.8%, which accords with the experiment (−0.8%).

## Discussion

Here we discuss the origin of the structural difference between BKT with tetragonal *P*4*mm* and BNT with rhombohedral *R*3*c*. We investigate the external pressure (*p*) dependence of the free energy *G* for the BKT and BNT cells by density functional theory (DFT) calculations, the details of which are described in Calculation Method and Supplementary Information II. For both the cells, the *R*3*c* structure is stabilized at a higher *p* while the *P*4*mm* one is at a lower *p*, because the *R*3*c* structure with octahedral rotations of *a*^−^*a*^−^*a*^−^ (Glazer notation) prefers a smaller cell volume (*V*) in the higher-*p* region. The equilibrium state (*p* = 0) was found in the *R*3*c* phase for the BNT cell with the *R*3*c*- *P*4*mm* boundary *p* (*p*_1_) at −2.21 GPa, while that appears in the *P*4*mm* phase with its *p* (*p*_2_) of 3.20 GPa. A partial substitution of K having a larger ionic radius (*r*_ion_) for Na increases the average *r*_ion_ on the A site and *V*; a negative chemical pressure caused by the K substitution increases the phase-boundary *p*.

Although we did not find an anomaly in the bond valence sum (BVS) as a function of *p* (Supplementary Fig. SI), the cation-O bonds show prominent features; especially, the Bi-O bonds exhibit a reconstruction across the phase boundary. In the centrosymmetric structure, Bi is surrounded by twelve O atoms. In the *R*3*c* structure, Bi is displaced along [111], leading to four different lengths: the shortest Bi-O1 (×3), followed by Bi-O2 (×3), Bi-O2* (×3) and Bi-O1* (×3), where asterisk (*) denotes a longer bond. Essentially, Bi-O1 is independent of *p* at ~0.240 nm, whose length is in good agreement with the experiments^48^. By contrast, Bi-O2 is lengthened when *p* decreases, leading to a smaller BVS of Bi. In the *P*4*mm* structure, the displacement of Bi along [001] results in three different lengths: the shorter four, the intermediate four (Bi-O1) and the longer four. Moreover, the tetragonal *P*4*mm* accommodates the markedly short Bi-O2 of ~0.225 nm.

**Figure 4:**
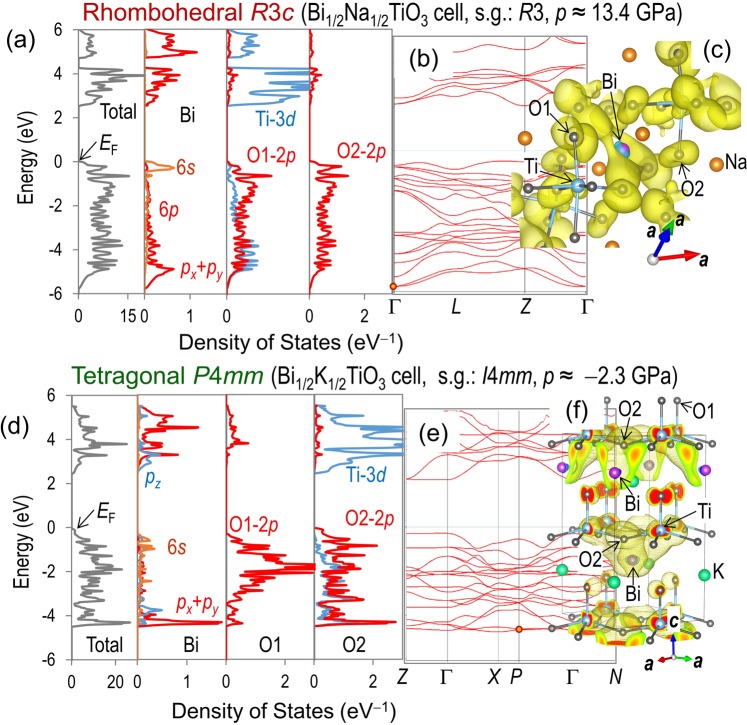
Density of states (DOS) calculations. DFT calculation results of (**a**–**c**) the *R*3*c* structure in the BNT cell at *p* = 13.4 GPa and (**d**–**f**) the *P*4*mm* structure in the BKT cell at *p* = −2.3 GPa; (**a**,**d**) partial DOS for Bi, O1 and O2 atom, (**b**,**e**) band structures, and (**c**,**f**) wave functions at the *k* points denoted by the dot marks in the band structures.

To elucidate the origin of the phase stability, we investigate the electronic structures at *p*~13.4 GPa and −2.3 GPa; the high *p* stabilizes the *R*3*c* structure (Fig. 4a–c) while the low *p* does the *P*4*mm* phase (Fig. 4d–f). The conduction band is formed primality by Ti-3*d*, and the valence band has a dominant contribution of O-2*p*. We note that the marked density of states (DOS) of not only Ti-3*d* but also Bi-6*p* appears in the valence band; especially the hybridized states of Bi-6*p* and O-2*p* determine the bottom of the valence band. At *p*~13.4 GPa in the BNT cell, the *R*3*c* phase features a dominant contribution of the Bi-6*p* (*p*_*x*_ + *p*_*y*_) derived states around the bottom with a minimum at −5.79 eV in the vicinity of the Γ point (the wavefunction is seen in Fig. 4c), which is lower by ~0.1 eV than the *P*4*mm* [Supplementary Fig. S2(a,b)]. We found that the stabilization of the *R*3*c* phase for BNT stems from the low-lying valence states arising from the Bi-6*p* and O-2*p* hybridization.

The similar feature is also seen in the BKT cell at *p*~−2.3 GPa; the mixed states of Bi-6*p* and O-2*p* dominate the bottom of the valence band. The maximal DOS of Bi-6*p* lies at ~−4.3 eV for the *P*4*mm* phase, which is higher than that (~−4.5 eV) for the *R*3*c* phase [Supplementary Fig. S2(c,d)]. However, the *P*4*mm* phase exhibits a markedly large DOS arising from the Bi-6*p* (*p*_*x*_ + *p*_*y*_) and O-2*p* in-plane hybridization (see the wavefunction shown in Fig. 4f), which is due to a small band dispersion in the entire Brillouin zone. Indeed, the atomic partial charge of Bi-6*p* is 0.89 for the *P*4*mm* phase, which is larger than 0.85 for the *R*3*c* phase. We found that the *P*4*mm* phase of BKT is stabilized by a large DOS of the Bi-6*p* (*p*_*x*_ + *p*_*y*_) states derived from the orbital interaction with O-2*p*.

In summary, we uncover the ferroelectric polarization and piezoelectric strain constant in the BKT-based single crystals grown by the high-*P*_O2_ TSSG process. These properties originate from a *P*_s_ of ~56 μC/cm^2^ with a *c*/*a* of ~1.6%; this *P*_s_ rivals that of PbTiO_3_ (70 μC/cm^2^). Our theoretical calculations show that the Bi-6*p* and O-2*p* hybridization at a moderately negative chemical pressure stabilizes the ferroelectric distortion in tetragonal symmetry. We could apply the high-*P*_O2_ process to other functional materials including Bi and/or K in bulk and film forms, here that we have developed high-quality BKT-based crystals by suppressing a defect formation reaction.

## Methods

### Experimental procedure

We prepared powders of (1 − *x*)BNT-*x*BKT (*x* = 30–35%) via solid-state reaction of the raw materials of Bi_2_O_3_ (99.99%), TiO_2_ (99.99%), Na_2_CO_3_ (99.99%), and K_2_CO_3_ (99.99%). These starting materials were mixed using ball milling with 100-μm beads and then calcined at 1,223 K for 4 h. The calcined powders were crushed by the ball milling and then calcined again at 1,423 K for 4 h to achieve a homogeneous solid solution.

For crystal structural anlyises, we performed time-of-flight (TOF) NPD measurements using a neutron powder diffractometer iMateria (BL20)^47^ at Japan Proton Accelerator Research Complex (J-PARC). NPD data in the *d* range of 0.05 < *d*/nm < 0.25 were collected with a high resolution Δ*d*/*d* = 0.16%. The crystal structure was refined by the Rietveld method with a computer software Z-Rietveld^49^.

We adopted the high-oxygen-pressure top-seeded solution growth (high-*P*o_2_ TSSG) method to obtain high-quality BNT–BKT single crystals, the details of which are described in refs. ^45,46,50^. 70%BNT-30%BKT (*x* = 30%) powders were mixed with a Bi_2_O_3_-KF flux at a weight ratio of BNT-BKT: Bi_2_O_3_ (99.99%): KF (99%) = 10: 10: 2. The mixture was soaked at over 1,430 K for 4 h in a Pt crucible rotated at 10 rpm, and then slowly cooled to approximately 1,400 K to form the solution. We used a BNT seed crystal obtained by a conventional flux method, which was set with a {001} plane normal to the melt surface. The seed rotated at 20 rpm counter to the crucible rotation was dipped into the solution, held at the same height for 1–4 h, and then slowly pulled out at a rate of 0.1–0.2 mm h^−1^ for over 20 h during cooling (<0.5 °C h^−1^). Eventually, a grown bulk crystal was detached from the solution and then slowly cooled to room temperature. The chemical composition of the grown crystal was analysed by inductively coupled plasma-atomic emission- spectrometry, indicating that the chemical composition is BNT–33%BKT with a composition deviation less than 1%.

The single crystals obtained were annealed at 1173 K for 10 h in air to remove mechanical stress induced during the crystal growth. The annealed crystals were cut along the {001} and {111} plane into plates with a thickness of 0.2 mm, and then gold electrodes were sputtered onto the cut surfaces. We measured polarization and leakage current of the crystal at 298 K using a ferroelectric test system (Toyo Corporation; Model 6252 Rev. B), and strain properties using a laser Doppler displacement meter.

### Calculation methods

DFT calculations were performed via the generalized gradient approximation^51^ with a plane wave basis set. The projector-augmented wave method^52^ was applied by the Vienna *ab initio* simulation package (VASP)^53^. We employed the gradient-corrected exchange-correlation functional of the Perdew-Burke-Ernzerhof revised for solids (PBEsol)^54^ and a plane-wave cut-off energy of 520 eV. The adopted mesh size of the *k*-point sampling grid was less than 5 nm^‒1^ for structural optimizations, 2.5 nm^−1^ for density-functional perturbation theory (DFPT) calculations. A rock-salt-like A-site ordering were adopted for constructing the Bi_1/2_Na_1/2_TiO_3_ and Bi_1/2_K_1/2_TiO_3_ cells^55,56^.

To obtain the Born effective charges, all the atomic positions were optimized in the Bi_1/2_Na_1/2_TiO_3_ and Bi_1/2_K_1/2_TiO_3_ cells under the constraints of the fixed lattice constants determined by the NPD analysis. Adopting a weighted average (mol %) of the Born effective charges (*Z*_eff_*) of the constituent atoms obtained in their respective Bi_1/2_Na_1/2_TiO_3_ and Bi_1/2_K_1/2_TiO_3_ cells, we estimated the averaged *Z*_eff_* of each atom in the BNT-BKT solid solutions. The calculations for the Bi_1/2_Na_1/2_TiO_3_ cell result in the following *Z*_eff_* values: 3.9 *e* for Bi, 1.1 *e* for Na, 6.1 *e* for Ti, −5.1 *e* for O_c_, and −1.7 *e* for O_a_, where *e* indicates the elementary charge of 1.602 × 10^−19^ C. We also found 4.0 *e* for Bi, 1.2 *e* for K, 6.0 *e* for Ti, −3.4 *e* for O_c_, and −2.3 *e* for O_a_ for the Bi_1/2_K_1/2_TiO_3_ cell. Adopting a weighted averaging of the Born effective charges in the respective cells on the mol% base, we obtain the averaged effective *Z*_eff_* of each atom in BNT–30%BKT: 2.6 *e* for Bi_0.50_Na_0.35_K_0.15_ (A-site atom), 6.1 *e* for Ti, −3.6 *e* for O_c_, and −2.3 *e* for O_a_.

To evaluate phase stability, we calculated the total energy (*U*) per ABO_3_ unit cell as a function of the cell volume (*V*) for the Bi_1/2_Na_1/2_TiO_3_ and Bi_1/2_K_1/2_TiO_3_ cells in *R*3*c* and *P*4*mm* symmetries, and then analyzed by the Murnaghan equation of state^57^:

1 $$U(V)={U}_{0}+\frac{{B}_{0}V}{{{B}^{{\rm{^{\prime} }}}}_{0}}[\frac{{({V}_{0}/V)}^{{{B}^{{\rm{^{\prime} }}}}_{0}}}{{{B}^{{\rm{^{\prime} }}}}_{0}-1}+1]-\frac{{B}_{0}{V}_{0}}{{{B}^{{\rm{^{\prime} }}}}_{0}-1},$$

where *U*_0_, *B*_0_, *B*_0_′, and *V*_0_ are the total energy, the bulk modulus and its first derivative with respect to the hydrostatic pressure (*p*) and *V* at *p* = 0. Since the free energy (*G*) is expressed as *G* = *U* + *pV*, we can obtain the relation between *G* and *p* using the fitting parameters in Eq. 1. The arrangement of the A-site atoms in the cells lowers the symmetry, i.e., the space group of the rhombohedral changes from *R*3*c* to *R*3 and that of the tetragonal from *P*4*mm* to *I*4*mm*. For simplicity, the higher symmetry is used to denote the space group throughout this paper.

## Supplementary information

Supplementary Information
