# Supplementary material for: Uncovering ferroelectric polarization in tetragonal (Bi1/2K1/2)TiO3–(Bi1/2Na1/2)TiO3 single crystals
Source: Sci Rep. 2019 Dec 17;9:19275. doi: 10.1038/s41598-019-55576-y (PMC6917808; doi:10.1038/s41598-019-55576-y)
Supplement: Supplementary file 1 — Supplementary Information [file 41598_2019_55576_MOESM1_ESM.pdf]

Uncovering ferroelectric polarization in $(Bi_{1/2}K_{1/2})TiO_3$-based single crystals

Yuuki Kitanaka, Yuji Noguchi*, and Masaru Miyayama

School of Engineering, The University of Tokyo, 7-3-1 Hongo, Bunkyo-ku, Tokyo 113-8656, Japan

*Address correspondence to ynoguchi@fmat.t.u-tokyo.ac.jp and yuji19700126@gmail.com.

TABLE OF CONTENTS

➢ Supplementary Information

I. Extended data for the Rietveld analysis

II. Extended data for the DFT calculations

➢ References in Supplementary Information

I. Extended data for the Rietveld analysis

Supplementary Table SI. Crystallographic data for BNT–30 %BKT at 295 K determined by the Rietveld refinements of the neutron powder diffraction data. The crystallographic data[1] of the BNT–9 %$BaTiO_3$ (BT) at 295 K are also shown for comparison.

	BNT-30 %BKT (295 K)	BNT-9%BT (295 K)
Radiation	Neutron (TOF)	Neutron (TOF)
Space group	*P*4*mm*	*P*4*mm*
a [nm]	0.38940 (1)	0.38925 (1)
c [nm]	0.39577 (1)	0.39580 (1)
c/*a* [–]	1.0164 (1)	1.0168 (1)
Δz (A-site) [–]	0.086 (2)	0.082 (2)
Δz (B-site) [–]	0.056 (2)	0.046 (2)

Supplementary Table SII. Fractional coordinates and equivalent anisotropic displacement parameters (the unit is 10^{-2} nm^2) of BNT–30%BKT at 295 K.

	x	*y*	*z*	U^{11}	U^{22}	U^{33}	U^{12}	U^{13}	U^{23}
$Bi_{0.50}Na_{0.35}K_{0.15}$	0	0	0	0.040(1)	0.040(1)	0.095(1)	0	0	0
Ti	1/2	1/2	0.471(1)	0.009(1)	0.009(1)	0.033(1)	0	0	0
O_c	1/2	1/2	−0.077(1)	0.033(1)	0.038(1)	0.014(1)	0	0	0
O_a	0	1/2	0.410(1)	0.003(1)	0.032(1)	0.050(1)	0	0	0

II. Extended data for the DFT calculations

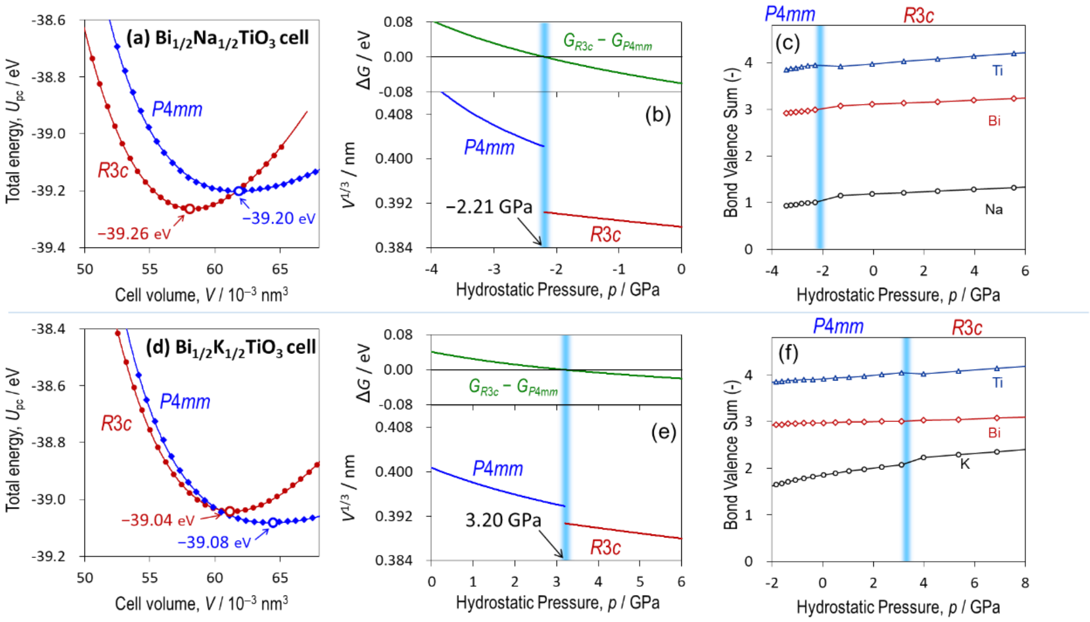

Supplementary Figure S1. DFT calculation data for (a–c) the $Bi_{1/2}Na_{1/2}TiO_3$ and (d–f) the $Bi_{1/2}K_{1/2}TiO_3$ cells; (a,d) the total energy U as a function of cell volume V, (b,e) the relations between free energy G, V and pressure p, along with (c,f) the variation in bond valence sum (BVS) as a function of p. Using the fitting parameters in Eq. S1, we obtain G_{R3c} for the rhombohedral $R3c$ and G_{P4mm} for the tetragonal $P4mm$ along with their difference, which is expressed as $\Delta G = G_{R3c} - G_{P4mm}$. For the BNT cell, the equilibrium state (p = 0) was found in the $R3c$ phase. In the higher-p region, ΔG becomes negative; the rhombohedral $R3c$ is stabilized at a smaller V. In contrast, in the lower-p region, ΔG becomes positive; the $P4mm$ phase is lower in free energy at a larger V. With decreasing p, the phase-boundary p (p_1) appears at ~ –2.21 GPa, and the $P4mm$ phase is stabilized. In reality, in the solid solutions, provided that Na with a smaller ionic radius (r_{ion}) is partly replaced by K with a larger r_{ion}, an average r_{ion} on the A site increases with increasing K content, which expands the unit cell, i.e., yields a larger V. Supposing that the system expands or shrinks by a chemical pressure that is controlled by the average r_{ion}, we can think that the phase transition is induced by a chemical pressure mediated through the V change: a negative pressure increases V and eventually triggers the phase transition from the $R3c$ to $P4mm$ phase at p_1.

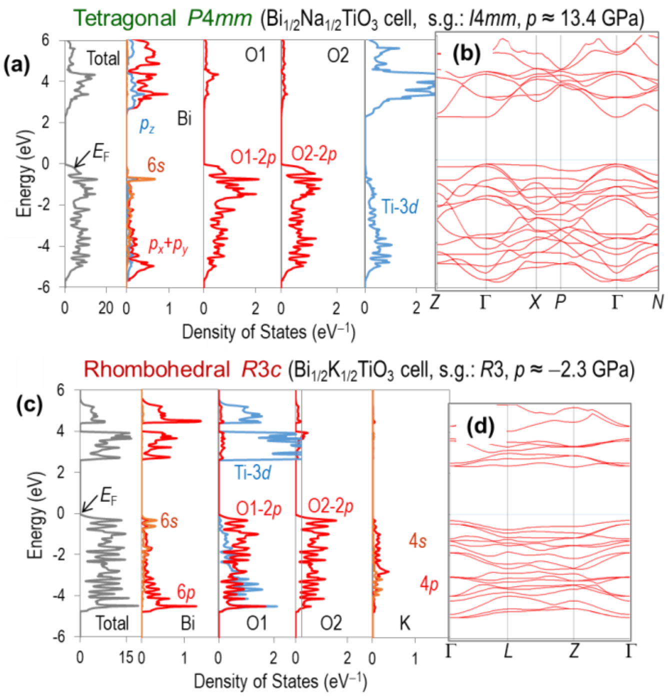

Supplementary Figure S2. Density of states (DOS) calculations of (a,b) the *P*4*mm* structure in the $Bi_{1/2}Na_{1/2}TiO_3$ cell at p = 13.4 GPa and (c,d) the *R*3*c* structure in the $Bi_{1/2}K_{1/2}TO_3$ cell at p = −2.3 GPa; (a,c) partial DOS for Bi, O1 and O2 atom, and (b,d) band structures.

References in Supplementary Information

1. Kitanaka, Y., Miyayama, M. & Noguchi, Y. Ferrielectric-mediated morphotropic phase boundaries in Bi-based polar perovskites. *Sci. Rep.* **9**, 4087 (2019).
